# Consumer Neuroscience and Digital/Social Media Health/Social Cause Advertisement Effectiveness

**DOI:** 10.3390/bs9040042

**Published:** 2019-04-18

**Authors:** Joanne M Harris, Joseph Ciorciari, John Gountas

**Affiliations:** Centre for Mental Health, Faculty of Health, Art and Design, Swinburne University of Technology, PO Box 218, Hawthorn 3122, Australia; jciorciari@swin.edu.au (J.C.); j.gountas@outlook.com (J.G.)

**Keywords:** consumer neuroscience, neuromarketing, EEG, public health digital/social media advertisements, social marketing, advertising effectiveness, action/challenge/emotion-based marketing, behavioural change

## Abstract

This research investigated the use of consumer neuroscience to improve and determine the effectiveness of action/emotion-based public health and social cause (HSC) advertisements. Action-based advertisements ask individuals to ‘do something’ such as ‘act’, ‘share’, make a ‘pledge’ or complete a ‘challenge’ on behalf of a brand, such as doing ‘something good, somewhere, for someone else’. Public health messages as noncommercial advertisements attempt to positively change behavioural intent or increase awareness. Australian health expenditure was $180.7 billion AUD (Australian dollars) in 2016/17 with $17 million AUD spent on government health campaigns. However, evaluation of health advertisement effectiveness has been difficult to determine. Few studies use neuroscience techniques with traditional market research methods. A 2-part study with an exploratory design was conducted using (1) electroencephalography (EEG) using a 64 channel EEG wet cap (n = 47); and (2) a Qualtrics online psychometric survey (n = 256). Participants were asked to make a donation before and after viewing 7 HSC digital/social media advertisements and logos (6 action/emotion-based; 1 control) to measure changes in behavioural intent. Attention is considered a key factor in determining advertising effectiveness. EEG results showed theta synchronisation (increase)/alpha desynchronisation (decrease) indicating attention with episodic memory encoding. sLORETA results displayed approach responses to action/emotion-based advertisements with left prefrontal and right parietal cortex activation. EEG and survey results showed the greatest liking for the ManUp action/emotion-based advertisement which used male facial expressions of raw emotion and vulnerability. ManUp also had the highest increased amount donated after viewing. Lower theta amplitude results for the International Fund for Animal Welfare (IFAW) action/emotion-based advertisement indicated that novel (possessing distinct features) rather than attractive/conventional faces were more appealing, while the rapid presentation of faces was less effective. None of the highest peak amplitudes for each ad occurred when viewing brand logos within the advertisement. This research contributes to the academic consumer neuroscience, advertising effectiveness, and social media literature with the use of action/challenge/emotion-based marketing strategies, which remains limited, while demonstrating the value in combining EEG and neuroscientific techniques with traditional market research methods. The research provides a greater understanding of advertising effectiveness and changes in behavioural intent with managerial implications regarding the effective use of action/challenge/emotion-based HSC communications to potentially help save a life and reduce expenditure on ineffectual HSC marketing campaigns.

## 1. Introduction

This research investigated how consumer neuroscience can improve the effectiveness of action/emotion-based health and social cause (HSC) digital/social media advertisements. ‘Action-based’ advertisements ask individuals to ‘do something’ such as ‘act, ‘share’, make a ‘pledge’ or complete a ‘challenge’ [1,2,3]. These include a request for something, such as uploading a photograph of oneself doing a particular activity [4]; or doing ‘something good, somewhere, for someone else’ [5]. A distinction is made between the recent development (since 2008) of this type of action/emotion-based HSC advertisements that differ from earlier ‘call to action’ advertisements that primarily asked individuals to make a donation. This study examined whether successful action/emotion-based HSC digital/social media advertisements were more effective than predominantly rational-based appeals.

A current marketing challenge is how to provide an idea that consumers will decide to interact or become involved with, in part addressed by the recent increase in action-based [6] or what can also be referred to as challenge marketing. With recent developments in marketing as a result of the influence of major technological developments and evolution of new media and digital formats [7] the long established AIDA (attention, interest, desire, action) model outlining the four stages consumption process [8] has been questioned by marketing industry professionals [1,2,6]. Ferrier suggests that the consumption process begins with action, from which an emotional connection with the brand will follow [6]. This coincides with the increasing development of action-based and in particular challenge marketing strategies such as ‘Dry July’ (2008) [9], ‘FebFast’ (2017) [10], or the 100 Day Challenge (2018) [11]. These action/challenge-based marketing campaigns ask people to give up certain substances such as alcohol (Dry July and FebFast), gambling (100 Day Challenge), and sugar, or pause doing/change any other activity, such as eating meat or reducing inactivity, in order to improve wellbeing (FebFast). Such action/challenge-based campaigns usually have an emotion rather than rational-based strategy focusing on benefits. Emotion-based strategies which attempt to build an emotional connection with consumers have largely dominated marketing in preference to prior rational-based approaches that provide consumers with numerous facts and figures [6]. Despite these marketing developments there are very few studies addressing action-based marketing strategies (see [1,6]).

Neuroscientific methods measure changes in neural electrical or metabolic activity [12] to study cognitive processes [13]. Neurological tools such as EEG can identify the driving forces behind an individual’s decision to become involved in HSC marketing campaigns that cannot be identified with traditional qualitative research methods such as self-report and focus groups [14,15]. Further, few consumer neuroscience studies and research designs use neuroscience techniques in combination with traditional marketing research methods such as those used in this research. The consumer neuroscience literature suggests there is a need for such an approach [16] with neuroscience techniques to be considered an additional market research technique rather than a replacement of traditional market research methods. As consumer neuroscience is an emerging discipline [17,18], there is a growing need and interest in the application of neurological quantitative research tools [12,19,20,21,22,23] for the development and analysis of digital/social media HSC advertisements [24,25].

Public health messages are noncommercial advertisements which attempt to change public behaviour [25], although commercial marketing practices are now commonly used in HSC communications [26]. Health campaigns aim to educate the public using health messages about risky behaviour while promoting healthier choices and encouraging ‘positive’ social conduct [25]. HSC communications or public service announcements [25] are also referred to as social marketing which uses marketing concepts to influence voluntary change in behaviour of target audiences in an attempt to improve societal health and well-being [26].

In 2013, $7.2 trillion USD (US dollars) was spent on global health [27]. Healthcare expenditure in European countries in 2016 was €233.9 billion euro in the UK, with Germany €350.2 billion, France €257.2 billion, Italy €150.2 billion, Spain €100.3 billion, Switzerland €74 billion, the Netherlands €72.8 billion, Sweden €50.9 billion, Belgium €42.4 billion, Austria €36.9, Norway €35.2 billion, Denmark €28.7, Poland €27.8 billion, Finland €20.5 billion, and Ireland €20.3 (figures include all health care providers) [28].

Australian government health expenditure was $180.7 billion AUD (Australian dollars) in 2016/2017 [29], while UK health expenditure was £143.7 billion in 2015–2016 [30]. UK government healthcare expenditure was £152.2 billion in 2016, while total UK healthcare expenditure was £191.7 billion (government and nongovernment), marking a 3.6% increase from £185.0 billion in 2015 [31].

Australia spent $17 million AUD on government health campaigns [32], not including advertising campaign expenditure below $250,000 AUD [32]. This marks an $8 billion AUD increase in expenditure from $172 billion AUD in 2013 [27]. The United States healthcare industry advertising expenditure in 2015 was $9.7 billion (increased by 11.3%) [33].

Despite the substantial global expenditure on health, healthcare, and health advertising, the evaluation and measure of effectiveness of health communications has been difficult to determine [34]. As a result, knowledge of key factors for the success of HSC marketing campaigns varies, and debate in the literature about advertising effectiveness and success factors remains divided.

Effectiveness of health communications has been defined as “the effect on knowledge, behavior or health outcomes” [35] measured by an intervention’s positive impact on health resulting in behavioural change [35]. Effectiveness has been identified as one of social marketing’s four Es: effectiveness, exaggeration, ethicality, and expensiveness [36]. Measures of advertising effectiveness include intrusiveness, as a gauge of attention and memory; persuasion, resulting in purchase intent [37]; attitudes (towards the ad) [37]; and engagement [38]. Research has found social marketing campaigns to have positive short-term effects, but these tend to decline mid to long term [26]. Therefore, the following hypothesis was proposed to address advertising effectiveness and changes in behavioural intent:

### Hypotheses

**Aim**: *Action/emotion-based marketing communications that ask individuals to ‘act’, ‘share’, ‘pledge’ or challenge’ are more effective than predominantly rational-based appeals.*

**Hypothesis** **1.** *Viewing action/emotion-based rather than rational-based advertisements increase donation amounts.*

Kong’s [39] ‘impression index’ uses memory and attention to determine the effectiveness of video commercials. The standard marketing decision-making process model involves four stages: (1) attention, (2) interpretation, (3) evaluation, and (4) memory [40]. The model includes working and long-term memory, with a distinction made between high and low involvement decision making [40]. However, research has shown that learning can occur with low levels of or no attention, suggesting that attention may not necessarily be the first stage of some decision-making processes [41]. Hence, in the increasingly fragmented digital media environment, such linear models of decision making may be considered obsolete in some instances.

Attention is considered an essential factor in achieving advertising effectiveness [42]. Attention is processed in the occipital, parietal, and right frontal regions [43]. Left hemispherical activation has been associated with memory [44] and long-term memory (LTM) processing [45]. Prefrontal cortex (PFC) processing has been associated with executive cognitive functions and processing such as planning, multitasking, social behaviour, personality display [46], and decision making [47,48,49,50].

EEG’s high temporal resolution allows it to accurately measure neural responses [22] and emotional valence [51,52,53]. Theta synchronisation/alpha desynchronisation has been shown to indicate attention and long-term episodic memory encoding [39,54]. Research has shown that asymmetrical theta increase/alpha decrease with hemispherical lateralisation indicates processing of pleasant (left)/unpleasant (right) emotions [55]. In particular, the left/right frontal cortex is associated with the like/dislike dichotomy of advertisements [56]. Left/right hemispherical lateralisation of approach (left)/withdraw (right) indicates positive/negative valence of emotions [52,53,57,58]. Further, psychophysiological measures of arousal (high and low) [15] operate as antecedents of advertising effectiveness [59]. A major limitation of EEG is the inability to identify spatial localisation [12]. However, sLORETA (low resolution brain electromagnetic tomography) [60] is able to identify cortical activation and corresponding Brodmann areas from EEG data using 3D head modelling [13]. Therefore, the following hypothesis was proposed to address attention and memory:

**Hypothesis** **2.** *Theta synchronisation (increase)/alpha desynchronisation (decrease) while watching action/emotion-based advertisements indicates participants are paying attention and an increase in episodic memory encoding.*

High interactivity increases attention and cognitive processing [61]. Interactive digital marketing has grown considerably due to the interactive and interconnected capabilities of Web 2.0 [62,63]. As a result, interactivity has become an intrinsic communication tool used on the internet [64]. Online digital/social media advertising enables viewers to choose to consume or interact in response to marketing communications [59]. Thus, consumers have become heavily involved and influential in terms of exchange in the marketing communications environment [62] and regularly engage with business and other consumers [65]. Interpersonal social interactions are considered fundamental in terms of encouraging individuals to adopt beneficial health behaviour changes, whereas mass media approaches are more suitable for generating awareness about health issues [66]. Thus, the internet as a hybrid channel has the potential to enable the implementation of behavioural change health interventions on a large scale [66]. Further, research has shown that voluntary rather than forced interactivity is an important factor to consider when considering the effect of interactivity [2,67,68].

Despite the growth of social media advertising [69] and increase of public health communications using social media, there remain a limited number of studies of public health campaigns using these channels [70,71]. Further, there is a shortage of publications regarding the use of social media [72] for the delivery of public health communications and interventions [71]. Consequently, social media user engagement, participation, and patterns of dissemination are not widely understood [72]. Therefore, social media health communication effectiveness and their use of social media vehicles and networks has been difficult to evaluate [71]. However, the effectiveness and level of engagement of digital/social media marketing campaigns is usually determined by quantitative measurement of likes, shares, comments, views and followers etc. [72]. Engagement is a field of investigation in itself; for further discussion see [73,74,75,76,77,78,79,80].

## 2. Materials and Methods

This study aimed to compare, inform, and triangulate results from EEG data and self-report measures (online survey data) to provide greater understanding of cognitive and decision-making processes involved in changes in behavioural intent in response to action/emotion-based HSC marketing communications. The combined use of consumer neuroscience (EEG) and traditional quantitative research methods (online survey) was the result of a recent review of the literature suggesting that neuroscience techniques are an additional tool, not a replacement research method, and should be used in combination, but that this rarely occurs in consumer neuroscience research [81].

Derived from four measures of advertising effectiveness discussed earlier, measures used for this research were (1) attention/memory (intrusiveness/engagement), (2) changes in donation amounts (influence/purchase intent), and (3) like/dislike of ads (attitudes). The changes in donation amounts were used to measure changes in behavioural intent, so that donation amounts themselves were not the primary measure.

As consumer neuroscience is a nascent field, the exploratory research design replicates approaches taken by established consumer neuroscience researchers (see [22,25,68,82,83]). The methodology used was based on gold standard studies [24,39,84] that investigated advertising effectiveness and included the use of an unrelated video as the control. Consumer neuroscience methodologies use neuroimaging techniques such as EEG, fMRI, eye-tracking, and biometric markers to evaluate advertising effectiveness. Often, consumer neuroscience research designs involve participants viewing different types of advertisements, depending on the research topic, while neuroscience techniques are used to record brain activity.

### 2.1. Method

Data were collected using two research methods, which involved (1) EEG recordings and (2) a Qualtrics online psychometric survey. The experiment used an exploratory design which involved 4 tasks, with 3 used to monitor changes in behavioural intent, followed by a post-task survey as the fourth. Ethics approval was obtained by the University Human Research Ethics Committee, SUHREC Project No: SHR Project 2016/234.

The experiment comprised 7 digital/social media advertisements (6 action/emotion-based, HSC advertisements and an unrelated rational-based advertisement that operated as a control). All advertisements were selected based on their degree of success, determined by marketing campaigns’ quantitative evaluation for consumer reach effectiveness, industry awards received, number of social media likes/shares generated, popularity, increase in sales, spin-offs (TV documentaries, case studies, and their adoption by leading business schools), celebrity endorsement and support, media attention, and coverage (see [3]).

The 7 advertisements selected for inclusion in the experiment were ManUp Campaign (2016); Unicef Tap Project: Put down your phone to help give clean water to kids (2016); DKMS, Help, I want to save a life (2012); Agilis Accounting (2014); United Nations World Humanitarian Day, Beyoncé – I was here (2012); Clinton Foundation, We’re not there yet (2015); and International Fund for Animal Welfare (IFAW), Tails for whales (2009). ManUp, Tap Project, and IFAW were categorised as having an action/emotion-based strategy in addition to being considered very successful advertisements. Agilis was categorised as a less successful, neutral/mediocre advertisement utilising a rational-based strategy and was therefore used as control variable. Clinton Foundation, DKMS, and United Nations used an emotion-based strategy and ranged from very successful (DKMS and United Nations) to less successful (Clinton).

### 2.2. Part 1: EEG study

EEG recruitment methods, exclusion criteria, sample size, experiment process details below.

Participant recruitment: Voluntary participation via posters, letterbox drops, Facebook, word of mouth, online platforms, university research experience program (REP) (academic credits granted).

Exclusion criteria: Participants were required to be 18 years of age and over and were excluded if they suffered from epilepsy, had a neurological or psychiatric condition, had had convulsions or head trauma and were taking medication for their condition.

Informed consent: All participants read and signed an informed consent form outlining the nature of the study, participant rights in terms of consent, withdrawal from the study, confidentiality, and ethics approval.

Sample size: A total of *n* = 47 adults from general population were recruited but reduced to *n* = 40 as 1 male and 6 female participants were subsequently excluded due to previous exposure to experiment research design and content, test subjects, and equipment issues. Final sample size comprised 13 (30%) males and 27 (70%) females. Participants’ ages ranged from 18–57 years (M = 27.2 years, SD = 10.003). Males were slightly older with a mean age of 28.77 years, with that of females being 26.74 years.

Experiment process: All participants completed informed consent and demographic details forms before participating in the EEG experiments. Every participant was seated in a dedicated darkened laboratory room with a 28-inch ViewSonic LED 1080p Full HD monitor (screen 25.5 inch, 118.5cm from floor to base of screen, standing 1 metre in front of participant). Participants viewed 7 digital/social media advertisements (6 action/emotion-based, 1 control) which included viewing the ad brands’ logos shown before and after the advertisements were screened, while EEG recorded (tasks 1–3). Presentation of logos and request for donations (tasks 1 and 3) were used to monitor changes in behavioural intent and donations made after viewing the advertisements. At the end of viewing, all advertising communications participants completed a short 7-question paper-based survey (task 4, EEG not recorded).

Participants wore a 64 channel EEG wet cap with electrodes placed according to the international 10/20 system [68,82]. EEG reference and ground electrodes used as references [68,82]. Impedance of electrodes was held below 5kΩ [68,82] on Compumedics Neuroscan SynAmps_RT_. EEG recorded on Compumedics Neuroscan Scan4.0 during baseline conditions (eyes open, closed, open left, right, up, and down), and while digital/social media communications were viewed (tasks 1–3 activation) [82].

Presentation of stimuli was randomised for each participant, varying the order for each task recorded automatically on a stim computer. Short breaks in viewing occurred prior to each task while eye tracking calibration and validation was reset.

**Task 1**: Participants were seated in front of a computer screen and keyboard and asked to place their left hand’s fingers on the Z, X, and C keys (to reduce muscle artefact in EEG recordings). Participants were then informed that after viewing a logo, they would be asked on screen to make a donation of $30, $15 or $0 dollars by pressing the Z (30 dollars), X (15 dollars) or C (0 dollars) keys. Participants then viewed 7 logos, after which they were asked on a grey screen with white text to donate $30 (Z), $15 (X) or $0 (C) dollars. The next stimuli appeared on screen once participants had pressed one of the three designated keys.

**Task 2**: Participants viewed 7 digital/social media advertisements but were not required to respond to the target stimulus nor do any typing/action on the keyboard.

**Task 3**: Repeated first task.

**Task 4**: Post-task survey (7 questions). EEG not recorded.

**Figure 1:**
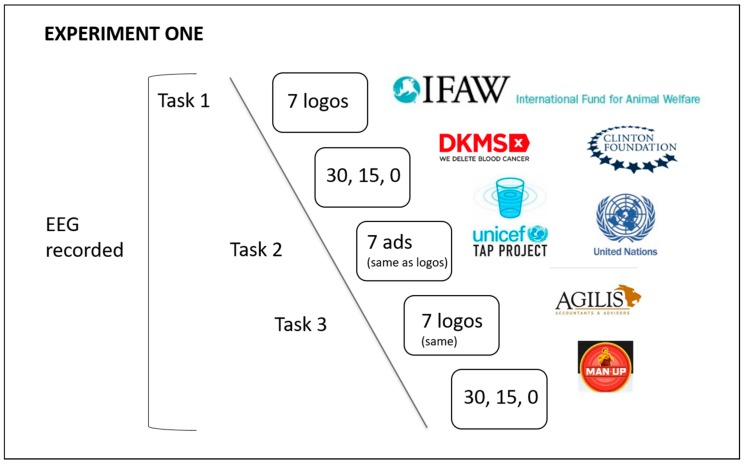
Experiment flow chart, tasks 1–3.

This experiment was created using PsychoPy2 Experiment Builder (v1.84.2) [85,86]. A graphic representation of the experiment flow chart tasks 1–3 is shown in Figure 1 below.

Brain Products’ Brain Vision Analyzer 2.1 software was used to clean data and analyse EEG recordings. Brain Vision Analyzer (BVA) uses the term segmentation when different sections of data are identified. The term epoch is used in neuroscience for the same process. As segmentation is a common term used in marketing in reference to the identification of a target market, the term epoch is used in this paper in preference to segmentation in order to reduce potential confusion.

Data cleaning was conducted in Excel, imported into IBM SPSS Statistics version 24. Statistical analysis of BVA data was conducted in SPSS and Excel. SPSS was used to conduct t-tests and ANOVA. Statistical power for EEG and online study was calculated using G*Power 3.1.9.2. All data files were processed to remove artefact and analysed according to the following protocol:

Brain Vision Analyzer: All files processed to have the same sampling rate. Bad channels were removed and replaced. Baseline correction was conducted (used in BVA to remove preset stimulus of each epoch). Eye blink artefact was not removed as data were very clean, and preference to include all data recorded due to the relative short length of each ad. An IIR Filter (zero phase shift Butterworth) was applied. Ad markers were identified in the EEG recording. The ad order was recorded on a stim computer used with stimulus markers located in EEG recordings to identify the beginning and end times of ads. Ads were identified individually in recordings and epoched for each ad separately. Fast Fourier transform and grand average were conducted.

Fast Fourier Transform: Fast Fourier transform (FFT) was conducted. FFT band was exported for all electrodes for theta, alpha, and beta bands. Band averages for each electrode were generated from FFT. Theta and alpha bands were extracted for each epoch. Band average data for each participant and each electrode were cleaned and sorted in Excel. Data was imported into IBM SPSS Statistics version 24. SPSS was used to conduct t-tests and ANOVA.

**Figure 2:**
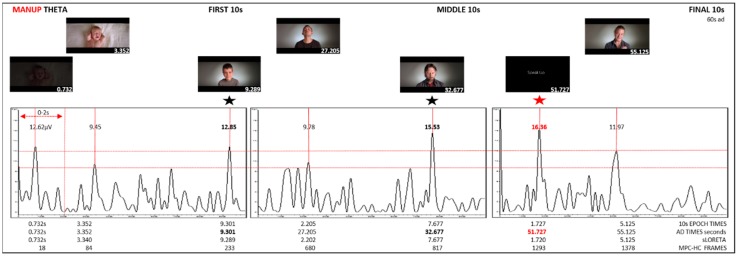
ManUp grand average theta from first, middle, and final 10 s epochs.

**Figure 3:**
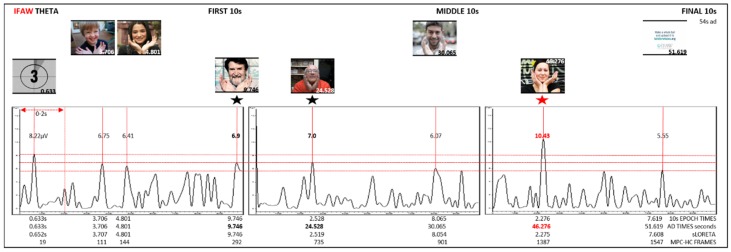
IFAW grand average theta from first, middle, and final 10 s epochs.

**Figure 4:**
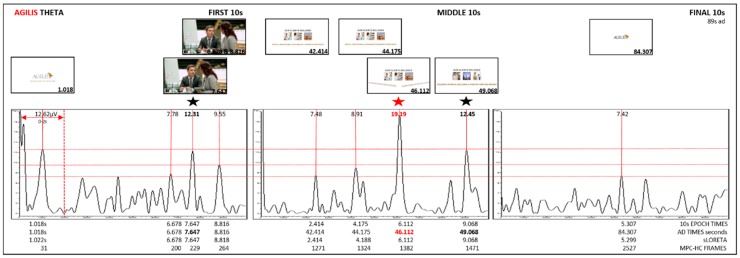
Agilis grand average theta from first, middle, and final 10-s epochs.

Grand Average: Epochs for the first, middle, and final 10 s created. Grand average was conducted for each epoch. Theta, alpha, and beta bands were extracted from the grand average. Amplitude peaks for each epoch were identified. The amplitude and time of highest peaks for theta and alpha were recorded in Excel. sLORETA maps for highest peaks were generated in BVA. Brain regions and Brodmann areas were identified in BVA. EEG times for each epoch were converted to ad times. MPC-HC X 64 was used to identify frames using the highest peak time in each epoch. Frames were recorded. Grand average graphs using 10 s EEG traces and corresponding frames were created (Figure 2, Figure 3 and Figure 4).

SPSS: A paired sample t-test (two-tailed) of the frontal and parietal electrodes from the FFT export averages of alpha and theta means was conducted for ManUp, IFAW and Agilis. The electrodes used were: FpZ, Fz, FcZ, left Fp1, AF3, F7, F5, F3, F1, FC3, FC1, FC5, P7, P5, P3, P1, PO7, PO5, PO3, and right Fp2, AF4, F2, F4, F6, F8, FC2, FC4, FC6, P2, P4, P6, P8, PO4, PO6, PO8, *n* = 40 (Table 1). Participants’ demographic data (age, gender, and education) were recorded in Excel. Descriptive statistics of demographic data were generated using the data analysis tool in Excel. The t-test results were exported to Excel, where they were cleaned and formatted. Theta mean average for each ad was used to calculate paired differences between ManUp (independent variable) and Agilis (dependent variable), and IFAW and Agilis.

G*Power [87,88,89]: Test family: t-test. Statistical test: Means: Difference between two dependent means (matched pairs). Type of power analysis: Post hoc: Compute achieved power—given α, sample size, and effect size. Input parameters: two-tailed. Effect size dz: dz = 0.5. α error probability: α = 0.5/0.8. Total sample size: (EEG *n* = 40). Output Parameters: α = 0.5, noncentrality parameter δ = 3.162, critical t = 2.023, df = 39, power (1-β error probability) = 0.869 (87%); α = 0.8, noncentrality parameter δ = 5.06, critical t = 2.023, df = 39, power (1-β error probability) = 0.999 (100%). Statistical power <0.80 was considered robust for behavioural science data [89,90,91].

### 2.3. Part 2: Qualtrics Psychometric Online Survey

The Qualtrics online psychometric survey replicated the EEG research design, except that presentation of the logos and digital/social media clips were not randomised, survey was anonymous, and due to time constraints, no additional demographic information was collected other than gender. The online survey comprised 23 parts (16 survey parts followed by a 7 question post-task survey) in addition to viewing 7 advertisements, which took in total approximately 15–30 min maximum to complete. The survey questions replicated the EEG research design in that participants were shown a brand logo and then asked to donate $30, $15 or $0 dollars (task 1), then this task was repeated (task 3) after participants had viewed the advertisements (task 2), in order to measure changes in behavioural intentions and decision making. The brief 7 question post-task survey questions (task 4) consisted of nominal, Likert scale, and multiple-choice questions to determine brand awareness, familiarity, prior knowledge/interaction, future intentions, and brand like/dislike (attitudes).

A total of *n* = 256 adults (70 males, 146 females, 40 unknown) participated in the online survey. A report was generated in Qualtrics using the finished surveys’ filter, *n* = 216 (70 males, 146 females). Data from the Qualtrics survey were then exported to IBM SPSS Statistics Version 24 and all incomplete survey responses excluded, *n* = 152 (53 males, 99 females). Survey sample size, exclusion criteria, recruitment methods and informed consent details are below.

Sample size: *n* = 256 (70 males, 146 females, 40 unknown); *n* = 152 (53 males, 99 females), 34.87% males, 65.13% females.

Exclusion criteria: Survey incomplete. Participation in related survey or EEG project.

Recruitment: Voluntary participation, word of mouth, Facebook posts, online platforms, university research experience program (REP) (academic credits granted).

Informed consent: All participants read and signed an informed consent form outlining the nature of study, participant rights in terms of consent, withdrawal from study, confidentiality, ethics approval, and given option to agree/disagree to participate.

## 3. Results

### 3.1. EEG Study

ManUp, IFAW, and Agilis ads were selected for EEG analysis based on online survey results, which showed ManUp to have the greatest increased change in total donation amount. Agilis had the lowest amount, with a decrease in total donations, and IFAW a neutral response with no change in the total donation amount before and after viewing the advertisements.

**Figure 5:**
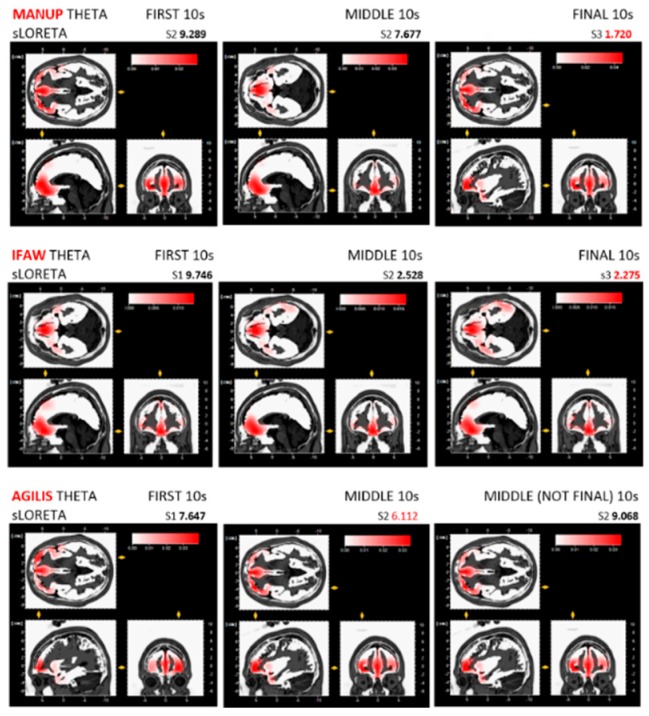
ManUp, Agilis, and IFAW sLORETA tomographic maps of three highest theta amplitudes from the first, middle, and final 10-s epochs (includes BVA sec times (not ad times) for 10-s epochs). *Agilis shows two sLORETA tomographic maps from the middle 10-s epoch, not final 10-s epoch, as three highest theta amplitudes occurred in the first and middle epochs, not the final epoch.

Results for ManUp, IFAW, and Agilis extracted grand average theta band from the first, middle, final 10 s epochs of EEG recordings show theta synchronisation (increase) with alpha desynchronisation (decrease) (Table 2). The corresponding video images for peak EEG theta averages are shown in Figure 2, Figure 3 and Figure 4. sLORETA (low resolution brain electromagnetic tomography) [60] maps generated in BVA show lateralised brain region activation of grand average synchronised theta/desynchronised alpha peaks for the ManUp, IFAW, and Agilis advertisements (Figure 5). The Brodmann areas from the sLORETA maps are shown in Table 3 (Appendix A).

Results from the paired t-test in SPSS of theta and alpha frontal and parietal electrode means from Brain Vision Analyzer 2.1 and grand average results for ManUp, Agilis, and IFAW showed theta synchronisation/alpha desynchronisation for frontal electrodes (Table 4; Table 5). EEG post hoc power analysis: *n* = 40, 2 tailed t-test, 87% power (>0.80) (α = 0.5, δ = 3.162, critical t = 2.023, df = 39); n = 40, 2 tailed t-test, 100% power (>0.80) (α = 0.8, δ = 5.06, critical t = 2.023, df = 39).

### 3.2. Online Survey

Using Qualtrics online survey results for donations made before and after viewing all 7 digital/social media advertisements (task 2) for the same brands as the logos viewed in task 1 and 3, the sum total of overall donations was calculated in Excel comparing changes in donation amounts.

**Figure 6:**
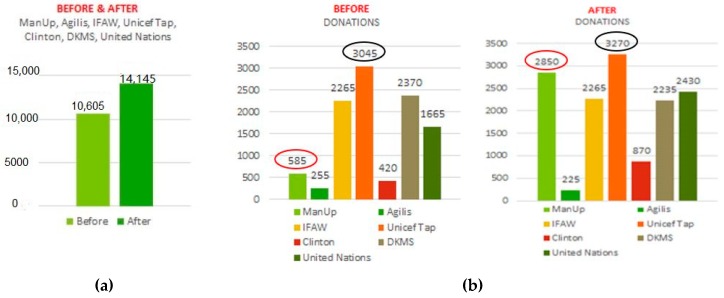
(**a**) Before and after changes in total donation amounts for all 7 digital/social media advertisements: ManUp, Agilis, IFAW, Unicef Tap Project, Clinton, DKMS, United Nations; (**b**) Breakdown of before and after changes in total donation amounts for each of 7 digital/social media advertisements.

Results indicated that total donations increased after viewing all 7 digital/social media advertisements (Figure 6a). A breakdown of changes in donations for each advertisement shows an increase in donations for ManUp ($585 to $2850 AUD), Unicef Tap Project ($3045 to $3270 AUD), United Nations ($1665 to $2430 AUD), and Clinton ($420 to $870 AUD), with no change in donation amount for IFAW ($2265 AUD before and after), and a decrease in donation amount for DKMS ($2370 to $2235 AUD) and Agilis (control) ($255 to $225 AUD) (see Figure 6b).

**Figure 7:**
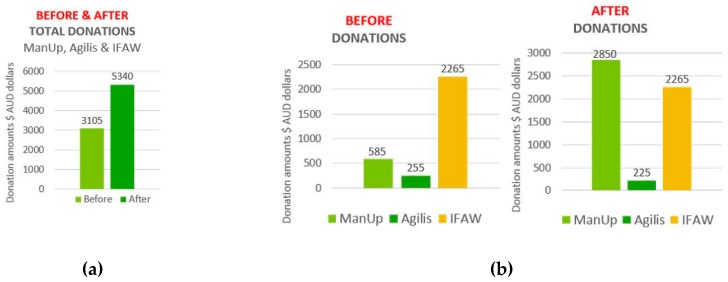
(**a**) Sum total amounts of changes in before and after donations for ManUp, Agilis, and IFAW; (**b**) Individual breakdown of changes in before and after donations amounts for ManUp, Agilis, and IFAW.

**Figure 8:**
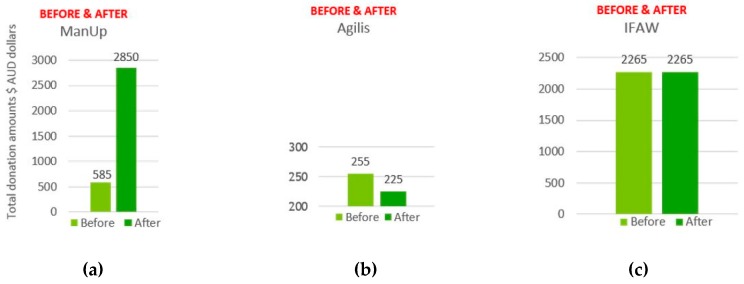
Before and after changes in donation amounts for (**a**) ManUp; (**b**) Agilis; and (**c**) IFAW.

The largest change and combined increase in total donations after viewing ManUp, Agilis, and IFAW advertisements show an increase of $2235 ($3105 to $5340 AUD) (Figure 7a). The highest amount of donations before and after (but not highest increase) was for the Unicef Tap Project ($3270) (Figure 7b). The highest amount showing the largest increase in total donations was shown for ManUp ($2280 increase) (Figure 7b and Figure 8a); the lowest donation amount and decrease of $30 was for Agilis (Figure 7b and Figure 8b), while IFAW total donations showed no change ($0) (Figure 7b and Figure 8c).

Two-tailed t-tests were conducted in SPSS for each advertisement comparing changes in donations made. The largest decrease in mean was for ManUp (0.987), with the smallest increase in mean for DKMS (−0.059), followed by Agilis (control) (−0.015), while IFAW showed no change (0.000) (Table 6).

Survey results showed the ManUp and Unicef Tap Project as the most equally liked ads. However, comparison of results on the like/dislike ordinal Likert scale showed ManUp to have the highest total liked, and the least disliked results. Unicef Tap Project as an action/emotion-based advertisement had the highest total donation amount and was liked as much as ManUp on the far point of the ‘like’ scale. However, while experiment tasks 1 and 3 asked participants to make a donation, the purpose of the research was to measure a change in participants’ behavioural intent as a result of viewing the advertisement, which was measured in terms of a change in donation amount. The research was not about whether participants decided to make a donation as a result of viewing the advertisement. Finally, although the difference between a greater preference for ManUp rather than Unicef Tap Project was minimal, the summed total of all like/dislike results showed ManUp to have the highest like and smallest dislike response and was therefore chosen for analysis rather than the Unicef Tap Project. However, the Unicef Tap Project warrants future analysis of results given that it is a more predominantly action rather than emotion-based advertisement in comparison to ManUp.

## 4. Discussion

There were significant changes in behavioural intent after viewing the advertisements shown in both the EEG and online studies. EEG study showed theta synchronisation/alpha desynchronisation in frontal electrodes for ManUp, IFAW, and Agilis. Only IFAW showed alpha desynchronisation in the parietal and parietal occipital electrodes (Table 4 and Table 5). The online survey showed an increase in donation amounts (change in decision-making/behavioural intent) for the most effective/liked action/emotion-based advertisement (ManUp), with a reduction in donation amounts for rational-based control (Agilis).

**Hypothesis 1 supported:** Viewing action/emotion-based rather than rational-based advertisements increased donation amounts. Based on online survey results, hypothesis 1 indicated that ManUp, the highest-ranking action/emotion-based advertisement, showed the greatest change in decision-making/behavioural intent (resulting in an increase in donation amounts). The EEG results showed that the use of raw emotion in an action/emotion-based ad (ManUp) that asked viewers to do something for the brand, i.e., SpeakUp in this instance, was the most effective approach, resulting in ad liking. EEG results indicated attention was an important factor as a measure of advertising effectiveness. EEG results identified the use of faces, in particular those showing raw emotion and vulnerability, as the most effective. While the use of novel rather than attractive or conventional faces was appealing, the use of faces being flashed in rapid succession (6 frames each at 29.97 frame rate) resulted in a neutral (neither like/dislike) response. Attractive is based on the perception of Western attractiveness stereotypes of beautiful faces being bilaterally symmetrical and average [92,93,94,95], with an approximate facial configuration that corresponds with the population, and are more easily processed [96], in addition to youthfulness and health [94,95]. Those considered novel faces in the IFAW advertisement are those with somewhat distinct features, such as a handlebar moustache or very large black sunglasses, rather than more conventional facial images without distinct features.

This research also showed that consumer neuroscience results differ from traditional market research techniques such as self-report in that EEG results showed a liking for the rational-based control ad (Agilis), whereas online self-report showed a strong dislike, resulting in a negative decision making/change in behavioural intent response with a reduction in donation amounts for this brand.

ManUp, as an action/emotion-based advertisement, used ‘raw emotion’ [97] with close-ups of male faces showing tears and vulnerability. Mulligan and Scherer suggest that no agreed definition of emotion exists across disciplines, but a distinction can be made between emotion (singular), classified as a group of affective processes, and emotions (plural) as specific types within that group [98]. Between disciplines, categorisation of basic emotions also varies [99]. Therefore, it would appear there is no agreed definition of ‘raw emotion’. Therefore, for this paper, ‘raw emotion’ refers to unmitigated expression of emotion without reservation or restraint and thus potentially exhibiting a degree of vulnerability or exposure.

ManUp aimed to encourage men to express their emotions in order to raise awareness about depression and male suicide. As a result of its success, a number of spin-offs occurred, including a documentary series featuring an acclaimed radio personality discussing his own experience with a male relative who died as a result of depression and suicide. Interestingly, facial images showing the highest EEG theta increase/alpha decrease responses have an averted, downward gaze (Figure 2). An averted rather than direct gaze has been associated with ‘avoidance-oriented emotions’ such as sadness [100]. Agilis (control) as a rational-based advertisement was a neutral, informative ad about accounting services offered. IFAW’s ‘Make a whale tail’ ad as an action/emotion-based advertisement asked viewers to send in a photo of themselves making a whale tail with their hands in support of anti-whaling. ‘Make a whale tail’ is the first social cause photo petition and one of the earliest highly successful action-based advertisements [3]. IFAW’s ad used a number of faces in rapid succession (only 6 frames each at frame rate of 29.97). Of note was that the most unusual (novel) rather than attractive (like) [101] faces in the IFAW ad showed the highest synchronised theta amplitudes. Further, despite the use of a large number of faces shown in rapid succession, this resulted in a relatively flat or indifferent overall EEG response, devoid of polarised high/lows (Figure 3). This was also reflected in IFAW online survey results, which showed $0.00 change in donations. These results suggest that while the use of faces has been shown to be appealing, the presentation method of facial imagery impacts viewers’ like/dislike/neutral response.

**Hypothesis 2 supported:** Theta synchronisation (increase)/alpha desynchronisation (decrease) in frontal electrodes while watching action/emotion-based advertisements indicated that participants were paying attention with an increase in episodic memory encoding.

Based on EEG results, hypothesis 2 indicated that participants were paying attention with increased episodic memory in varying degrees during particular sections of each advertisement. EEG results achieved 87% power post hoc (>0.80, *n* = 40, 2 tailed t-test, α = 0.5,) with 100% power post hoc (>0.80, α = 0.8). EEG t-test results indicated an increase in theta and decrease in alpha in the left and right hemisphere electrodes for ManUp, Agilis, and IFAW advertisements. Frontal electrodes showing theta synchronisation/alpha desynchronisation for all 3 advertisements were FpZ and Fz; left hemisphere Fp1, AF3, F7, F5, F3, and F1; and right hemisphere Fp2, AF4, F2, F4, F6, and F8. Only IFAW showed increases in theta and decreases with alpha in the parietal lobe left P7, P5, P3, P1, PO7, PO5, and PO3 and right electrodes P2, P4, P6, P8, PO4, PO6, and PO8 (Table 5).

ManUp’s highest theta amplitude for theta synchronisation/alpha desynchronisation occurred predominantly in the left hemisphere, whereas Agilis and IFAW’s highest peak occurred in the medial brain region (Figure 5, Table 3). Davidson’s circumplex model of affect divides basic emotions into pleasant/unpleasant and high/low states of arousal [102]. Only ManUp’s highest amplitude for theta synchronisation/alpha desynchronisation occurred in the left hemisphere, indicating a positive approach (like) response. Further, the highest amplitude image for ManUp as an action-emotion based approach was the call to ‘SpeakUp’ (Figure 2). Of note, none of the highest peak amplitudes for each ad occurred on viewing the brand logo at all, or in the final 10-s epoch. While placement of the brand logo at the end of an advertisement remains a popular marketing communications strategy, some research has shown that digital advertising is more effective if shown at the beginning [103] or for the duration of an advertisement, in preference to pulsing, which involves briefly presenting a brand logo a number of times throughout [42]. Recent academic and industry conference presentations also indicated that presenting the brand logo consistently throughout [104] and at the beginning of a digital advertisement [105] is more effective that showing the brand logo at the end.

sLORETA tomographic maps for the three highest peaks occurring in the first, middle or final 10-s epochs (Figure 5) showed activation in Brodmann areas 10 (BA10) and 11 (BA11). Located in the ventromedial PFC (vmPFC), Brodmann area 10 (BA10) [47] has been associated with personal relevance and engagement [106]. Attention and facial recognition have been shown to activate BA10 and 11 [82], shown in highest theta amplitudes for ManUp (BA10), IFAW, and Agilis (BA11) (Table 2; Table 3). Processing of faces occurs in the middle lateral fusiform gyrus [107] (fusiform face area, BA37) with unfamiliar faces processed in the right hemisphere [108]. Activation of the left middle temporal gyrus has been associated with empathy and processing of faces of well-known/famous identities [109]. During the first 10 s, BA10 was activated in the left hemisphere in the medial frontal gyrus for ManUp and the right hemisphere in the middle frontal gyrus for Agilis. ManUp’s synchronised theta amplitude peaks showed left hemisphere activation and attention to faces, while IFAW showed attention to faces and a well-known Australian identity (Figure 2, Figure 3 and Figure 5). BA11 in the middle frontal gyrus was activated with neither substantial left nor high hemisphere dominance for the IFAW advertisement (Figure 5). BA40 situated in the right supramarginal gyrus is actuated during decision making [47] which occurred for all three ads (Appendix A).

## 5. Conclusions

There are significant theoretical implications for the synthesis of concepts from the neuroscience and marketing disciplines. This research showed that action/emotion-based marketing communications that ask individuals to ‘act’, ‘share’, ‘pledge’ or challenge’ are more effective than predominantly rational-based appeals. Viewing action/emotion-based rather than rational-based advertisements resulted in a positive change in decision making with increased donation amounts and greater liking. ManUp as the highest ranking/most liked action/emotion-based advertisement had the greatest increase in donation amounts based on survey results. EEG results showed it was also the most liked advertisement.

Theta synchronisation (increase)/alpha desynchronisation (decrease) while watching action/emotion-based advertisements indicated that participants were paying attention with an increase in episodic memory encoding. The highest theta peak amplitude occurred for rational-based advertisement Agilis (Table 2, Figure 2, Figure 3 and Figure 4). The greatest liking occurred for the action/emotion-based approach using male facial expressions showing raw emotion and vulnerability with ManUp’s highest theta peak amplitude occurring in the final 10-s epoch when viewers were asked to act (‘SpeakUp’, Figure 2). Of note, none of the highest theta peak amplitudes for each ad occurred when viewing brand logos during the advertisement.

Limitations of the study were that despite a general population being used, this included participants from a student population. This was due to the recruitment methods, which included the use of university poster drops, Facebook, and word of mouth, in addition to a university research experience program (REP) where students received some academic course credit for participating. Both EEG and online survey studies had a female gender bias partly due to the researchers predominantly responsible for recruitment being female and that the REP program used for recruitment is available for participation by psychology students. Given that, in total, there are 28,667 female registered psychologists in Australia compared to 7236 males and 3 intersex/indeterminate [110], it is reasonable to conclude that university psychology courses tend to attract a much higher number of female than male students. Of note was a male bias amongst participants whose data were excluded from the online survey due to incompletion.

Additional limitations included that the funds donated were hypothetical rather than participants’ actual funds, so that individuals may behave differently if using their own funds. However, use of the REP program precluded financial payment for student participants. Further, ad lengths varied and, in some instances, could be considered too long (ranging from approximately 30 s to 2 min), which may have caused boredom amongst some participants. This was due to the nature of the study and selection of action/emotion-based ads, which are a relatively recent phenomenon (since 2008) and therefore limited in number. Further, the data collection occurred in a lab setting rather than a natural viewing environment, although advertisements were presented as closely as possible to a normal viewing situation.

Consumer neuroscience is considered an emerging discipline. Consumer neuroscience literature suggests there is a need for neuroscience techniques to be used in combination with traditional marketing research methods. As a relatively recent marketing communications’ strategy, action/emotion-based and social media advertising research remains limited.

This research contributes to the academic consumer neuroscience literature and evaluation of social media, advertising effectiveness, and action/emotion/challenge-based marketing strategies literature. Further, this research demonstrates how EEG and neuroscientific techniques can be combined with traditional market research methods and therefore has significant managerial implications regarding the analysis and future design of effective HSC marketing communications.

In summary, EEG highest theta peak amplitude occurred in the final 10-s epoch when viewing a tag line asking viewers to act. None of the EEG highest theta peak amplitudes for each ad occurred when viewing brand logos during the advertisement. Online survey results showed a positive change in decision-making/behavioural intent for the most effective/liked action/emotion-based advertisement, with a strong dislike and negative change in decision-making/behavioural intent for the rational-based control. However, EEG results showed liking for the rational-based advertisement, but self-report showed a strong dislike, resulting in a negative decision-making/behavioural intent response. The highest survey ad liking and greatest change in decision-making/behavioural intent occurred with action/emotion-based advertisement that asked viewers to do something for the brand, and used ‘raw emotion’. EEG results also showed that the use of raw emotion in an action/emotion-based ad was the most effective strategy resulting in ad liking. EEG results identified the use of faces, in particular those showing raw emotion and vulnerability, as the most effective. Further, novel (possessing distinct features) rather than attractive/conventional faces were more appealing, but the use of faces being flashed in rapid succession resulted in a neutral (neither like/dislike) response. These results suggest that while the use of faces has been shown to be appealing, the presentation method of facial imagery impacts viewers’ like/dislike/neutral response.

This study provides a greater understanding of advertising effectiveness of action/emotion-based HSC communications and contributes to marketing theory and consumer neuroscience development to potentially help to save a life and reduce expenditure on ineffectual HSC marketing campaigns.

## Figures and Tables

**Table 1:** Electroencephalography (EEG) electrodes used for the paired sample t-test.

Brain Region/Lobe	Central Electrodes	Left Hemisphere Electrodes	Right Hemisphere Electrodes
anterior frontal	FpZ	Fp1, AF3	Fp2, AF4
frontal	Fz	F7, F5, F3, F1	F2, F4, F6, F8
fronto-central	FCZ	FC3, FC1	FC2, FC4
left frontal temporal		FC5	FC6
parietal		P7, P5, P3, P1	P2, P4, P6, P8
parieto-occipital		PO7, PO5, PO3	PO4, PO6, PO8

**Table 2:** ManUp, International Fund for Animal Welfare (IFAW), and Agilis grand average theta and alpha amplitudes µV (from brand vision analyser (BVA)) for first, middle, and final 10-s epochs.

Ad	Grand Average Amplitudes µV	0–2 s	FIRST 10 s			MIDDLE 10 s			FINAL 10 s	
ManUp	theta average ↑ µV	12.85	9.45	12.85	X	9.78	15.53	X	16.36 * BA10, LH	11.97
	alpha average ↓ µV	0.535	1.87	0.728	X	0.180	0.0139	X	0.837	1.19
IFAW	theta average ↑ µV	8.22	6.75	6.41	6.9	7.0	6.07	X	10.43 * BA11, M	5.55
	alpha average ↓ µV	0.618	0.261	0.150	0.102	0.560	0.152	X	1.24	0.833
Agilis	theta average ↑ µV	12.62	12.31	9.55	X	7.48	8.91	19.19 * BA11, M	7.42	X
	alpha average ↓ µV	0.272	2.65	0.793	X	0.262	1.19	1.24	0.572	X

* highest theta peaks, Brodmann area (BA), left/right hemisphere (LH/RH), medial (M)).

**Table 3:** Brodmann areas from sLORETA tomographic maps for three highest amplitudes occurring in the first, middle or final 10-s epochs (Appendix A).

Advertisement 10 s Epochhs	Brodmann Area (BA)	Lobe	Greater Left or Right Hemisphere (LH/RH) Activation/Dominance	Brain Region: Frontal Gyrus
**FIRST 10 s (S1)**				
ManUp	BA10	Frontal	LH	Medial
IFAW	BA11	Frontal	neither substantial L/RH dominance	Medial
Agilis	BA10	Frontal	RH	Middle
**MIDDLE 10 s (S2)**				
ManUp	BA11	Frontal	neither substantial L/RH dominance	Medial
IFAW	BA11	Frontal	neither substantial L/RH dominance	Medial
Agilis	BA11	Frontal	LH	Medial
**FINAL 10 s (S3)/MIDDLE 10 s (S2) for Agilis** *				
ManUp	BA10	Frontal	LH	Middle
IFAW	BA11	Frontal	neither substantial L/RH dominance	Medial
Agilis	BA11	Frontal	LH	Medial

* Third highest amplitude occurred in the middle, not the final 10-s epoch for Agilis.

**Table 4:** Summary of 2-tailed t-test results of paired differences for frontal, parietal, and parietal–occipital regions showing theta/alpha synchronisation/desynchronisation (Alpha ↓ Theta ↑) (Appendix A).

2 TAILED T-TEST	ELECTRODES	N = 40	MEAN ALPHA ↓ THETA ↑			
PAIRS	Paired Differences (theta-alpha average)	Paired Mean	Paired Mean	Paired Mean	Paired Mean Differences	Paired Mean Differences
		ManUp	IFAW	Agilis	ManUp - Agilis	IFAW - Agilis
	**LEFT**					
Pair 1	FpzTH _*_ - FpzAPH ^†^	1.239	7.666	8.023	0.556	−0.356
Pair 3	FzTH - FzAPH	8.578	0.921	1.474	−0.234	−0.552
Pair 4	Fp1TH - Fp1APH	8.603	7.584	7.921	0.682	−0.337
Pair 5	AF3TH – AF3APH	6.123	5.067	5.551	−5.075	−0.483
Pair 6	F7TH - F7APH	2.175	1.574	2.773	−0.599	−1.200
Pair 7	F5TH - F5APH	2.153	1.591	2.453	−0.300	−0.862
Pair 8	F3TH - F3APH	2.200	1.376	1.945	0.254	−0.569
Pair 9	F1TH - F1APH	2.196	1.319	1.916	0.279	−0.598
	**RIGHT**					
Pair 13	Fp2TH - Fp2APH	8.211	7.237	7.707	0.504	−0.470
Pair 14	AF4TH - AF4APH	5.316	4.460	5.294	0.022	−0.835
Pair 15	F2TH - F2APH	0.590	0.315	1.134	−0.543	−0.818
Pair 16	F4TH - F4APH	0.742	0.579	1.353	−0.611	−0.774
Pair 17	F6TH - F6APH	0.671	0.652	1.833	−1.162	−1.181
Pair 18	F8TH - F8APH	1.243	2.016	2.982	−1.739	−0.966

* TH: Theta band; ^†^ APH: Alpha band.

**Table 5:** EEG electrodes showing increase in theta and decrease in alpha averages.

Advertisement	Brain Region/Lobe	Central Electrodes	Left Hemisphere Electrodes	Right Hemisphere Electrodes
ManUp, IFAW & Agilis	anterior frontal	FpZ,	Fp1, AF3	Fp2, AF4
ManUp, IFAW & Agilis	frontal	Fz	F7, F5, F3, F1	F2, F4, F6, F8
IFAW only	parietal		P7, P5, P3, P1	P2, P4, P6, P8
IFAW only	parieto-occipital		PO7, PO5, PO3	PO4, PO6, PO8

**Table 6:** Before and after mean, standard deviation, variance, and significance from the online survey for all 7 digital/social media advertisements.

Advertisement	Before		After		Variance		Sig.
N = 153 *	Mean	SD	Mean	SD	Mean	SD	
Unicef Tap Project	1.67	0.742	1.58	0.723	0.98	0.723	0.096
United Nations	2.27	0.78	1.94	0.771	0.333	−0.009	0.000
Agilis	2.89	0.373	2.90	0.358	−0.013	−0.015	0.707
DKMS	1.97	0.823	2.03	0.778	−0.059	−0.045	0.358
ManUp	2.75	0.532	1.76	0.698	0.987	0.166	0.000
IFAW	2.01	0.752	2.01	0.761	0.000	0.009	1.000
Clinton	2.82	0.436	2.62	0.618	0.196	0.563	0.000

* *n* = 153 not 152 as online survey data exported into Excel for all completed surveys used for analysis was 153, but an additional participant was excluded in SPSS due to rapid survey completion and repeat response indicating participant had not viewed digital advertisements and had responded to each question by pressing the same key.

## Note

This research was part of a three-part study where personality, eye-blink, and eye-tracking data were also collected. The second and third studies replicate the research design of the first study. These data will be used for comparative analysis and future research into the use of consumer neuroscience to evaluate the effectiveness of HSC marketing communications.

## Abbreviations

AC	Anterior Cingulate
BA	Brodmann Areas
IPL	Inferior Parietal Lobule
LH	Left Hemisphere
M(C)	Medial (central)
MFG	Medial Frontal Gyrus
MidFG	Middle Frontal Gyrus
OG	Orbital Gyrus
RH	Right Hemisphere

## Appendix A

**Table A1:** IFAW, Agilis three highest theta amplitudes from the first, middle, and final 10-s epochs (includes BVA times, ad times, and Brodmann areas (BA)) used for ad images of peak theta averages shown in Figure 2, Figure 3, Figure 4 and Figure 5 and Table 2; Table 3.

**AD**	**AD TIME**												
**MANUP**	**60 s**		**AMP**	**BVA TIME**	**AD TIME 10 s (10,000 ms)**	**MPC-HC**		**sLORETA MAP**	**BA**	**HEMI-SPHERE**	**LOBE**	**BRAIN REGION**	**PEAK**
EPOCH	THETA BAND	LOR	uV	Posn sec	AD S1: 0:00–10:00	FRAME	uAmm2	TALAIRACH COORDINATES					
S1 0–10 s		0.732	12.85	0.732	0.732	18	0.174	X = −3, Y = −60, Z = 57	BA7		Parietal	Precuneus	
		3.34	9.45	3.352	3.352	84	0.0218	(X = −3, Y = −52, Z = −6)	BA10		Frontal	MFG	
		9.289	12.85	9.301	9.301	233	0.0271	(X = −3, Y = −52, Z = −6)	BA10	LH	Frontal	MFG	highest
S2 25–35 s		2.202	9.78	2.205	27.205	680	0.0191	(X = −3, Y = 38, Z = −20)	BA11		Frontal	MFG	
		7.677	15.53	7.677	32.677	817	0.0393	(X = −3, Y = 38, Z = −20)	BA11	M(C)	Frontal	MFG	highest
S3 50–60 s		1.72	16.36	1.727	51.727	1293	0.0461	(X = −38, Y = 52, Z = −6)	BA10	LH	Frontal	MidFG	highest
		5.125	11.97	5.125	55.125	1378	0.0231	(X = −3, Y = 38, Z = −20)	BA11		Frontal	MFG	
**IFAW**	**54 s**		**AMP**	**BVA TIME**	**AD TIME**	**MPC-HC**		**sLORETA MAP**	**BA**	**HEMI-SPHERE**	**LOBE**	**BRAIN REGION**	**PEAK**
EPOCH	THETA BAND	LOR	uV	Posn sec	AD 0:00–10:00	FRAME	uAmm2	TALAIRACH COORDINATES					
S1 0–10 s		0.652	8.22	0.633	0.633	19	0.0233	(X = −3, Y = 52, Z = −6)	BA10		Frontal	MFG	
		3.707	6.75	3.706	3.706	111	0.0195	(X = −3, Y = 45, Z = −20)	BA11		Frontal	OG	
		4.801	6.41	4.801	4.801	144	0.0156	(X = −3, Y = 38, Z = −20)	BA11		Frontal	MFG	
		9.746	6.9	9.746	9.746	292	0.0132	(X = -3, Y = 38, Z = −20)	BA11	M(C)	Frontal	MFG	highest
S2 22–32 s		2.519	7	2.532	24.532	735	0.0169	(X = −3, Y = 38, Z = −20)	BA11	M(C)	Frontal	MFG	highest
		8.054	6.07	8.065	30.065	901	0.0147	(X = −3, Y = 52, Z = 1)	BA10		Limbic	AC	
		9.232	5.74	9.232	31.232	936	0.0208	(X = 60, Y = −39, Z = 29)	BA40		Parietal	IPL	
S3 44–54 s		0.493	5.06	0.491	44.491	1333	0.0455	(X = 60, Y = −39, Z = 29)	BA40		Parietal	IPL	
		2.275	10.43	2.276	46.276	1387	0.0183	(X = −3, Y = 38, Z = −20)	BA11	M(C)	Frontal	MFG	highest
		4.999	5.55	4.999	48.999	1469	0.0473	(X = 60, Y = −39, Z = 29)	BA40		Parietal	IPL	
		7.608	5.76	7.619	51.619	1547	0.0171	(X = −3, Y = 45, Z = −20)	BA11		Frontal	OG	
**AGILIS**	**89 s/1:29 m**		**AMP**	**BVA TIME**	**AD TIME**	**MPC-HC**		**SLORETA MAP**	**BA**	**HEMI-SPHERE**	**LOBE**	**BRAIN REGION**	**PEAK**
EPOCH	THETA BAND	LOR	uV	Posn sec	AD S1 0:00–10:00	FRAME	uAmm2	TALAIRACH COORDINATES					
S1 0–10 s		1.022	12.62	1.018	1.018	31	0.0297	(X = −3, Y = 45, Z = −20)	BA11		Frontal	OG	
		7.647	12.31	7.647	7.647	229	0.039	(X = 32, Y = 59, Z = −6)	BA10	RH	Frontal	MidFG	highest
		8.818	9.55	8.816	8.816	264	0.0191	(X = −3, Y = 38, Z = −20)	BA11		Frontal	MFG	
S2 40–50 s		2.414	7.48	2.414	42.414	1271	0.0139	(X = −3, Y = 38, Z = −20)	BA11		Frontal	MFG	
		4.188	8.91	4.175	44.175	1324	0.0237	(X = −3, Y = 45, Z = −20)	BA11		Frontal	OG	
		6.112	19.19	6.112	46.112	1382	0.0497	(X = −3, Y = 38, Z = −20)	BA11	M(C)	Frontal	MFG	highest
		9.068	12.45	9.068	49.068	1471	0.0341	(X = −38, Y = 52, Z = −6)	BA10		Frontal	MidFG	
S3 79–89 s	1:29	5.299	7.42	5.307	84.307 = 1:24:307	2527	0.016	(X = −3, Y = 52, Z = −6)	BA10	M(C)LH/RH	Frontal	MFG	highest

**Table A2:** Two tailed t-test theta average results for ManUp, IFAW, and Agilis showing Alpha ↓ Theta ↑.

**MANUP THETA ALPHA**		**Paired Samples T-Test**		**Paired Differences (2-tailed)**						
	**THETA-ALPHA**	**Mean**	**Alpha ↓ Theta ↑**	**Std. Deviation**	**Std. Error Mean**	**95% Confidence Interval of Difference**		**t**	**df**	**Sig.**
	**Frontal Electrodes**					**Lower**	**Upper**			**(2-tailed)**
Pair 1	FzmuTH* - FzmuALPH†	1.239	Alpha ↓ Theta ↑	11.550	1.826	−2.455	4.933	0.679	39	0.501
Pair 3	FpzmuTH - FpzmuALPH	8.578	Alpha ↓ Theta ↑	5.942	0.939	6.678	10.479	9.131	39	0.000
Pair 4	Fp1muTH - Fp1muALPH	8.603	Alpha ↓ Theta ↑	6.036	0.954	6.673	10.534	9.015	39	0.000
Pair 5	AF3muTH - AF3muALPH	6.123	Alpha ↓ Theta ↑	8.101	1.281	3.532	8.714	4.780	39	0.000
Pair 6	F7muTH - F7muALPH	2.175	Alpha ↓ Theta ↑	9.369	1.481	−0.822	5.171	1.468	39	0.150
Pair 7	F5muTH - F5muALPH	2.153	Alpha ↓ Theta ↑	9.738	1.540	−0.962	5.267	1.398	39	0.170
Pair 8	F3muTH - F3muALPH	2.200	Alpha ↓ Theta ↑	10.494	1.659	−1.156	5.556	1.326	39	0.193
Pair 9	F1muTH - F1muALPH	2.196	Alpha ↓ Theta ↑	11.290	1.785	−1.415	5.807	1.230	39	0.226
Pair 13	Fp2muTH - Fp2muALPH	8.211	Alpha ↓ Theta ↑	6.009	0.950	6.290	10.133	8.643	39	0.000
Pair 14	AF4muTH - AF4muALPH	5.316	Alpha ↓ Theta ↑	8.253	1.305	2.676	7.955	4.074	39	0.000
Pair 15	F2muTH - F2muALPH	0.590	Alpha ↓ Theta ↑	11.535	1.824	−3.099	4.279	0.324	39	0.748
Pair 16	F4muTH - F4muALPH	0.742	Alpha ↓ Theta ↑	10.749	1.700	−2.696	4.180	0.437	39	0.665
Pair 17	F6muTH - F6muALPH	0.671	Alpha ↓ Theta ↑	9.105	1.440	−2.241	3.583	0.466	39	0.644
Pair 18	F8muTH - F8muALPH	1.243	Alpha ↓ Theta ↑	8.218	1.299	−1.385	3.872	0.957	39	0.345
**IFAW THETA ALPHA**		**Paired Samples T-Test**		**Paired Differences (2-tailed)**						
	**THETA-ALPHA**	**Mean**	**Alpha ↓ Theta ↑**	**Std. Deviation**	**Std. Error Mean**	**95% Confidence Interval of Difference**		**t**	**df**	**Sig.**
	**Frontal electrodes**					**Lower**	**Upper**			**(2-tailed)**
Pair 1	FpzifTH - FpzifALPH	7.666	Alpha ↓ Theta ↑	8.781	1.388	4.858	10.475	5.522	39	0.000
Pair 3	FzifTH - FzifALPH	0.921	Alpha ↓ Theta ↑	12.858	2.033	−3.191	5.033	0.453	39	0.653
Pair 4	Fp1ifTH - Fp1ifALPH	7.584	Alpha ↓ Theta ↑	8.294	1.311	4.931	10.236	5.783	39	0.000
Pair 5	AF3ifTH - AF3ifALPH	5.067	Alpha ↓ Theta ↑	10.143	1.604	1.824	8.311	3.160	39	0.003
Pair 6	F7ifTH - F7ifALPH	1.574	Alpha ↓ Theta ↑	10.461	1.654	−1.772	4.919	0.951	39	0.347
Pair 7	F5ifTH - F5ifALPH	1.591	Alpha ↓ Theta ↑	10.829	1.712	−1.873	5.054	0.929	39	0.359
Pair 8	F3ifTH - F3ifALPH	1.376	Alpha ↓ Theta ↑	11.709	1.851	−2.368	5.121	0.743	39	0.462
Pair 9	F1ifTH - F1ifALPH	1.319	Alpha ↓ Theta ↑	12.455	1.969	−2.664	5.302	0.670	39	0.507
Pair 13	Fp2ifTH - Fp2ifALPH	7.237	Alpha ↓ Theta ↑	8.833	1.397	4.412	10.062	5.182	39	0.000
Pair 14	AF4ifTH - AF4ifALPH	4.460	Alpha ↓ Theta ↑	10.381	1.641	1.140	7.780	2.717	39	0.010
Pair 15	F2ifTH - F2ifALPH	0.315	Alpha ↓ Theta ↑	12.283	1.942	−3.613	4.244	0.162	39	0.872
Pair 16	F4ifTH - F4ifALPH	0.579	Alpha ↓ Theta ↑	11.256	1.780	−3.021	4.179	0.325	39	0.747
Pair 17	F6ifTH - F6ifALPH	0.652	Alpha ↓ Theta ↑	9.616	1.520	−2.423	3.727	0.429	39	0.670
Pair 18	F8ifTH - F8ifALPH	2.016	Alpha ↓ Theta ↑	10.830	1.712	−1.448	5.479	1.177	39	0.246
Pair 22	P7ifTH - P7ifALPH	0.816	Alpha ↓ Theta ↑	13.042	2.062	−3.355	4.987	0.396	39	0.694
Pair 23	P5if - P5ifALPH	0.884	Alpha ↓ Theta ↑	14.553	2.301	−3.771	5.538	0.384	39	0.703
Pair 26	PO7if - PO7ifALPH	1.684	Alpha ↓ Theta ↑	13.501	2.135	−2.634	6.002	0.789	39	0.435
Pair 27	PO5if - PO5ifALPH	1.645	Alpha ↓ Theta ↑	13.634	2.156	−2.715	6.006	0.763	39	0.450
Pair 28	PO3ifTH - PO3ifALPH	1.859	Alpha ↓ Theta ↑	13.171	2.082	−2.353	6.071	0.893	39	0.378
Pair 29	P2ifTH - P2ifALPH	0.109	Alpha ↓ Theta ↑	15.614	2.469	−4.885	5.103	0.044	39	0.965
Pair 30	P4ifTH - P4ifALPH	1.394	Alpha ↓ Theta ↑	14.853	2.349	−3.356	6.144	0.594	39	0.556
Pair 31	P6ifTH - P6ifALPH	2.557	Alpha ↓ Theta ↑	13.711	2.168	−1.828	6.942	1.179	39	0.245
Pair 32	P8ifTH - P8ifALPH	3.392	Alpha ↓ Theta ↑	14.629	2.313	−1.286	8.071	1.466	39	0.151
Pair 33	PO4ifTH - PO4ifALPH	3.418	Alpha ↓ Theta ↑	13.815	2.184	−1.001	7.836	1.565	39	0.126
Pair 34	PO6ifTH - PO6ifALPH	3.586	Alpha ↓ Theta ↑	13.669	2.161	−0.785	7.958	1.659	39	0.105
Pair 35	PO8ifTH - PO8ifALPH	3.301	Alpha ↓ Theta ↑	13.260	2.097	−0.940	7.541	1.574	39	0.123
**AGILIS THETA ALPHA**		**Paired Samples T-Test**		**Paired Differences (2-tailed)**						
	**THETA-ALPHA**	**Mean**	**Alpha ↓ Theta ↑**	**Std. Deviation**	**Std. Error Mean**	**95% Confidence Interval of Difference**		**t**	**df**	**Sig.**
	**Frontal electrodes**					**Lower**	**Upper**			**(2-tailed)**
Pair 1	FpzagTH - FpzagALPH	8.023	Alpha ↓ Theta ↑	6.205	0.981	6.038	10.007	8.177	39	0.000
Pair 3	FzagTH - FzagALPH	1.474	Alpha ↓ Theta ↑	10.590	1.674	−1.913	4.860	0.880	39	0.384
Pair 4	Fp1AagTH - Fp1agALPH	7.921	Alpha ↓ Theta ↑	6.180	0.977	5.945	9.897	8.106	39	0.000
Pair 5	AF3agTH - AF3agALPH	5.551	Alpha ↓ Theta ↑	7.925	1.253	3.016	8.085	4.429	39	0.000
Pair 6	F7agTH - F7agALPH	2.773	Alpha ↓ Theta ↑	6.658	1.053	0.644	4.903	2.635	39	0.012
Pair 7	F5agTH - F5agALPH	2.453	Alpha ↓ Theta ↑	7.747	1.225	−0.025	4.930	2.003	39	0.052
Pair 8	F3agTH - F3agALPH	1.945	Alpha ↓ Theta ↑	9.353	1.479	−1.046	4.937	1.315	39	0.196
Pair 9	F1agTH - F1agALPH	1.916	Alpha ↓ Theta ↑	10.193	1.612	−1.343	5.176	1.189	39	0.242
Pair 13	Fp2agTH - Fp2agALPH	7.707	Alpha ↓ Theta ↑	6.085	0.962	5.761	9.653	8.011	39	0.000
Pair 14	AF4agTH - AF4agALPH	5.294	Alpha ↓ Theta ↑	7.682	1.215	2.837	7.751	4.359	39	0.000
Pair 15	F2agTH - F2agALPH	1.134	Alpha ↓ Theta ↑	10.152	1.605	−2.113	4.380	0.706	39	0.484
Pair 16	F4agTH - F4agALPH	1.353	Alpha ↓ Theta ↑	8.879	1.404	−1.486	4.193	0.964	39	0.341
Pair 17	F6agTH - F6agALPH	1.833	Alpha ↓ Theta ↑	6.958	1.100	−0.392	4.058	1.666	39	0.104
Pair 18	F8agTH - F8agALPH	2.982	Alpha ↓ Theta ↑	7.180	1.135	0.686	5.278	2.627	39	0.012
